# Cryopyrin‐associated periodic syndrome with inflammatory bowel disease: A case study

**DOI:** 10.1002/jgh3.12523

**Published:** 2021-03-09

**Authors:** Karen N Raymond, Jonathan E D Martin

**Affiliations:** ^1^ School of Biological Sciences, The University of Adelaide Adelaide South Australia Australia; ^2^ Faculty of Health and Medical Sciences, The University of Adelaide Adelaide South Australia Australia

**Keywords:** anakinra, colitis, inflammation, inflammasome, infliximab

## Abstract

Cryopyrin‐associated Periodic Syndrome (CAPS) is a rare, genetic autoinflammatory condition associated with NLRP3 gene mutations, causing upregulated innate immunity. CAPS manifests as systemic inflammation, causing a constellation of symptoms on a clinical spectrum of phenotypical severity: Familial Cold Autoinflammatory Syndrome being the mildest, Muckle‐Wells Syndrome moderate, and Neonatal Onset Multisystem Inflammatory Disease the most severe, with phenotype spectrum overlap. The treatment in Australia for CAPS is interleukin‐1 blockade with receptor antagonist, anakinra. We describe the case of a 46‐year‐old female with CAPS who presented to the emergency department with severe abdominal pain. Sigmoidoscope revealed severe colitis with deep ulceration, which did not respond to hydrocortisone and azathioprine and was ultimately resolved by infliximab rescue therapy, maintained in combination with anakinra.

## Graphical abstract

Cryopyrin‐Associated Periodic Syndrome (CAPS) is a rare, autoinflammatory condition, caused by NLRP3 gene mutations and managed by interleukin 1 blockade. Here, a gastrointestinal manifestation with CAPS was successfully treated with anti‐TNF infliximab, maintained with anakinra.

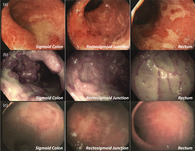

## Introduction

Cryopyrin‐associated Periodic Syndrome (CAPS) is a rare, genetic autoinflammatory condition associated with mutations in the NLRP3 gene that result in NLRP3 inflammasome‐mediated hyperactivation of proinflammatory cytokines interleukin 1β and interleukin 18. Systemic inflammation results in a constellation of symptoms on a clinical spectrum of phenotypical severity, which has defined Familial Cold Autoinflammatory Syndrome (FCAS) as the mildest, Muckle‐Wells Syndrome (MWS) as moderate, and Neonatal Onset Multisystem Inflammatory Disease (NOMID) as the most severe, with some phenotype overlap on this spectrum.^1^ Incidence is estimated at 1–3 per million; however, the multisystemic nature of CAPS means patients experience significant delays in diagnosis or misdiagnosis.

Symptoms are generally described as nonpruritic urticaria‐like rash (with cold exposure activation in FCAS), fever, arthralgia, myalgia, headaches, fatigue, and ocular involvement for all CAPS. Migraine, sensorineural hearing loss, and increased risk of amyloidosis occur in MWS and NOMID. Bone (especially knee) deformities and neurological impairment occur in NOMID.^1^ While inflammatory bowel disease has not been widely reported to be a feature of CAPS, NLRP3 has been implicated in inflammatory bowel disease.^2^ Evidence suggests that anti‐tumor necrosis factor (TNF) therapy for CAPS patients may be effective where interleukin 1 (IL‐1) blockade alone does not completely manage symptoms.^1^ However, concerns have been raised about potential harm from this combination.

## Case Report

A 46‐year‐old female with CAPS and previously diagnosed ulcerative proctitis presented to emergency with severe abdominal pain. She reported having spent nine nights in another hospital, from where she had been discharged and spent two nights at home with a high level of pain. Her CAPS mutation, A439V, has been described as both FCAS and MWS on the CAPS spectrum of disease severity. She had been diagnosed in May 2016 and was commenced on 100 mg daily subcutaneous anakinra, increasing to 200 mg after 7 months, which was ongoing. The patient suggested a first‐time flu vaccine 5 weeks earlier as a possible trigger for this episode. She had also only recovered from suspected influenza 1 week prior to being administered the vaccine. Influenza is known to induce IL‐1β and IL‐18 via NLRP3 inflammasome activation.

**Figure 1:**
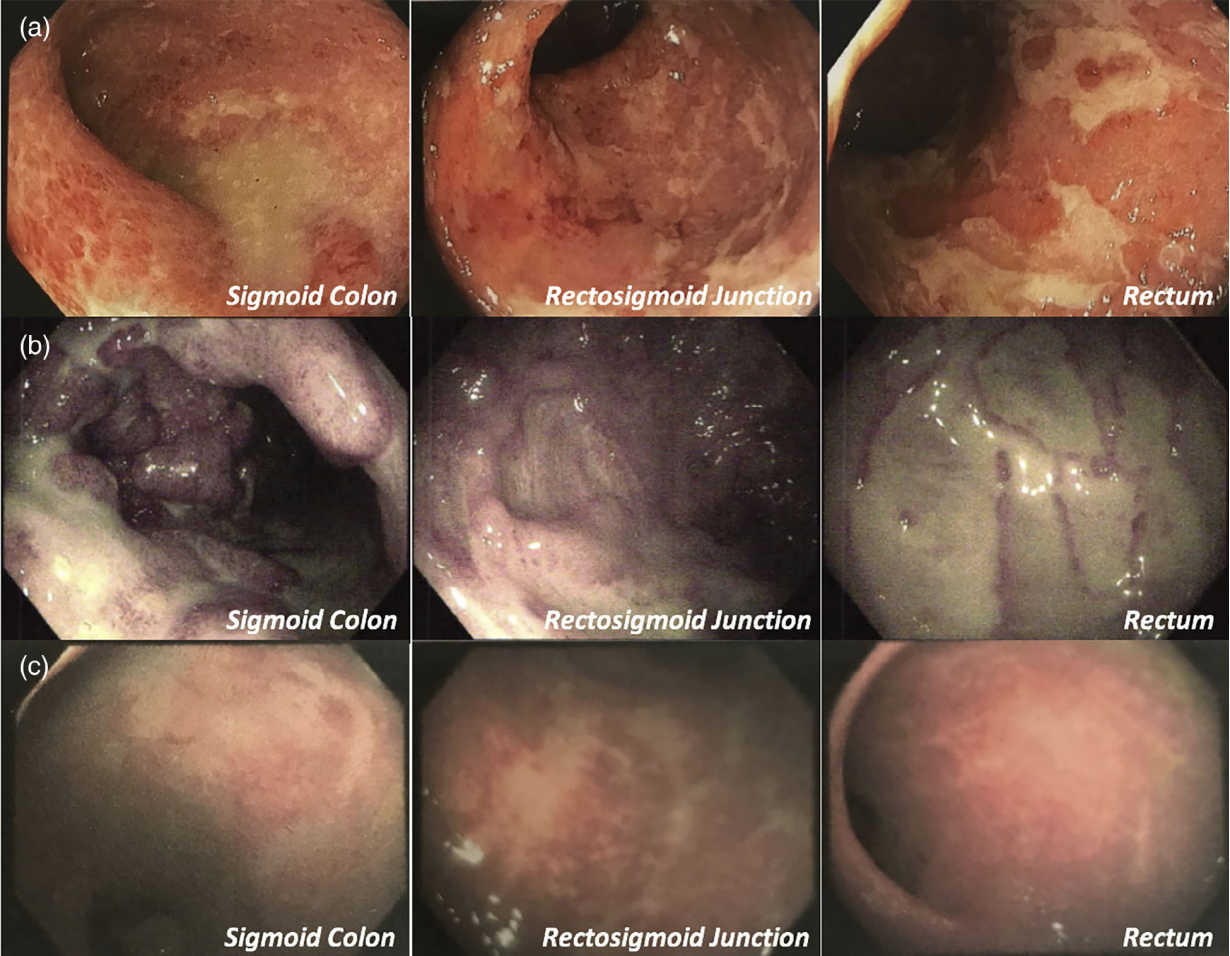
(a) Flexible sigmoidoscopy on initial presentation in June 2019 shows severe colitis to the extent of 45 cm from the anal verge on examination, with diffuse severely erythematous, ulcerated, granular mucosa with shallow and deep ulceration observed (Mayo 3); (b) flexible sigmoidoscopy in July 2019 after steroid treatment with azathioprine shows multiple deep ulcers found in the rectum, sigmoid, and descending colon (not shown) with exposure of the muscle layer and pseudopolyp formation; (c) flexible sigmoidoscopy in October 2019 following three infliximab infusions shows linear scars throughout the sigmoid and rectum, and a diffuse area of moderately erythematous and vascular‐patterned decreased mucosa was found in the rectosigmoid colon.

Her previous sigmoidoscope (Fig. 1a) revealed severe colitis to the extent of 45 cm from the anal verge on examination, with diffuse severely erythematous, ulcerated, granular mucosa with shallow and deep ulceration observed (Mayo 3; Mayo score is a disease activity index for ulcerative colitis where 0 is normal and 3 is severe). Intravenous (IV) hydrocortisone treatment was commenced, along with 50 mg of azathioprine. On discharge, after 9 days in the hospital, her C‐reactive protein (CRP) was 52 mg/L, and she was given oral prednisolone. After 2 days, she was readmitted to a different hospital with worsening pain that was not responding to 50 mg of tramadol hydrochloride.

## Management and Outcome

On her second hospital admission, the patient's CRP was 122.5 mg/L. She was restarted on IV hydrocortisone and immediately placed on an elemental diet, which she was able to sustain for 9 days. Azathioprine dosing was increased to 100 mg daily. Over the next 25 days, she required three potassium infusions, one iron infusion, and one blood transfusion. Sigmoidoscopy (Fig. 1b) revealed further deterioration, and infliximab rescue therapy was initiated, requiring her to cease anakinra due to contraindication of these treatments.^1^

After 10 days, the patient, a biomedical science student in her final year of study, resumed her anakinra medication at half her normal dose, only revealing this action 4 days after recommencement. She cited difficulty managing her CAPS symptoms, particularly in the hospital environment where exposure to air conditioning vents and inability to control room temperature were triggering factors. The patient had been able to obtain independent advice from two international CAPS experts with regard to her situation. She referred to recently published literature suggesting that anti‐TNF therapy may be indicated for CAPS patients whose symptoms are incompletely managed with IL‐1 inhibitors.^1^

Her response to infliximab was good, but CRP remained high. On resuming anakinra, her CRP came down to 6 mg/L within 24 h, and she was discharged 5 days later on prednisolone (60 mg, decreasing by 5 mg per week), azathioprine 150 mg, vitamin A, vitamin D, resprim forte, and tapentadol 100 mg SR for pain management with endone 5 mg additionally as needed. The azathioprine dose was readjusted to 100 mg and then stopped 8 weeks after discharge as the patient reported ongoing nausea in response to this medication.

Sigmoidoscopy at 11 weeks after final discharge (Fig. 1c) showed that the mucosa looked much improved from previous examinations, appearing intact with no deep ulcerations. Linear scars were seen throughout the sigmoid and rectum, and a diffuse area of moderately erythematous and vascular‐patterned decreased mucosa was found in the rectosigmoid colon. The patient reported being on no pain medication. At 3 and 6 months, her CRP was <1 mg/L, and at 6 months, all other blood work had returned to the normal range.

## Discussion

Due to the rarity of CAPS, expert advice in Australia is limited. There is a lack of safety data available with regard to the administration of anakinra, an interleukin‐1 receptor antagonist, in combination with infliximab, an anti‐TNF antibody. The primary concern is the resultant immunosuppression, leaving the patient vulnerable to infection. However, in this case, the patient required both therapies for a successful outcome. While anakinra is the primary treatment for CAPS in Australia, it has been shown to have limited efficacy in some cases. Interleukin‐1 inhibition has been demonstrated to result in increased TNF.^1^

In addition, interleukin‐18 is hyperactivated in CAPS patients through the formation of the NLRP3 inflammasome. Interleukin‐18 has been identified as a key regulator of intestinal inflammation through its differential effects on mucosal lymphocytes. Evidence suggests that interleukin‐18 plays a dual role in gastrointestinal mucosa, protective in an acute inflammatory response but damaging in long‐term chronic inflammation.^3^ The effects of interleukin‐18 in the specific context of CAPS and gastroenteric symptoms is an area where more research is needed that could be beneficial to inflammatory bowel disease generally. This patient had an atypical presentation of ulcerative colitis, with the absence of bloody diarrhea and presence of severe pain as the dominant features. Rather than ulcerative colitis, this could instead be an inflammatory bowel disease (IBD) associated with the patient's CAPS condition. Biopsies were not consistent with vasculitis.

The hyperactivation of innate immune signaling pathways in CAPS patients means that the specific immune‐suppressive treatments could be considered normalizing, rather than suppressing, in these patients. The problem remains the lack of specific biomarkers such that dosage continues to be largely guesswork and guided by clinical symptoms reported by the patient. With variation in CAPS disease phenotypes and severity among patients, even within the same family groups, it is difficult to formulate a consistent approach to treatment. In this case, the patient was highly educated in her own rare disease, which ultimately helped inform treating physicians.
